# Supplementary material for: Multicomponent Lipid Nanoparticles for RNA Transfection
Source: Pharmaceutics. 2023 Apr 20;15(4):1289. doi: 10.3390/pharmaceutics15041289 (PMC10141487; doi:10.3390/pharmaceutics15041289)
Supplement: Supplementary file 1 [file pharmaceutics-15-01289-s001.zip › pharmaceutics-2280590-supplementary_vb.pdf]

pharmaceutics

Supplementary Materials: Multicomponent Lipid Nanoparticles for RNA Transfection

Nataliya Gretskaya, Mikhail Akimov, Dmitry Andreev, Anton Zalygin, Ekaterina Belitskaya, Galina Zinchenko, Elena Fomina-Ageeva, Ilya Mikhalyov, Elena Vodovozova and Vladimir Bezuglov

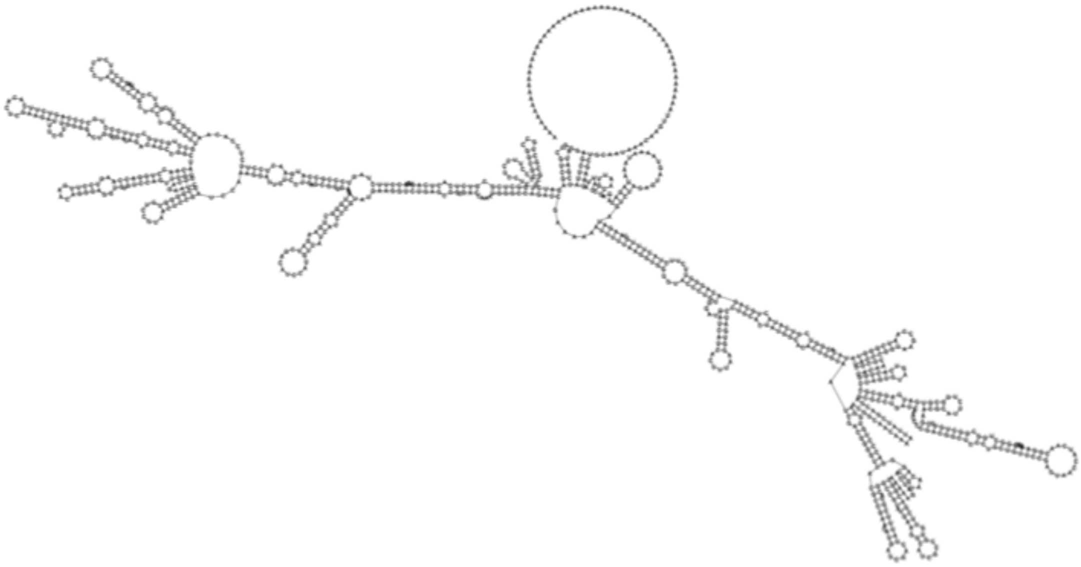

Figure S1. Model of mRNA second structure generated with ViennaRNA Package 2.0. Condition: minimum free energy prediction according to Turner, 2004 model.

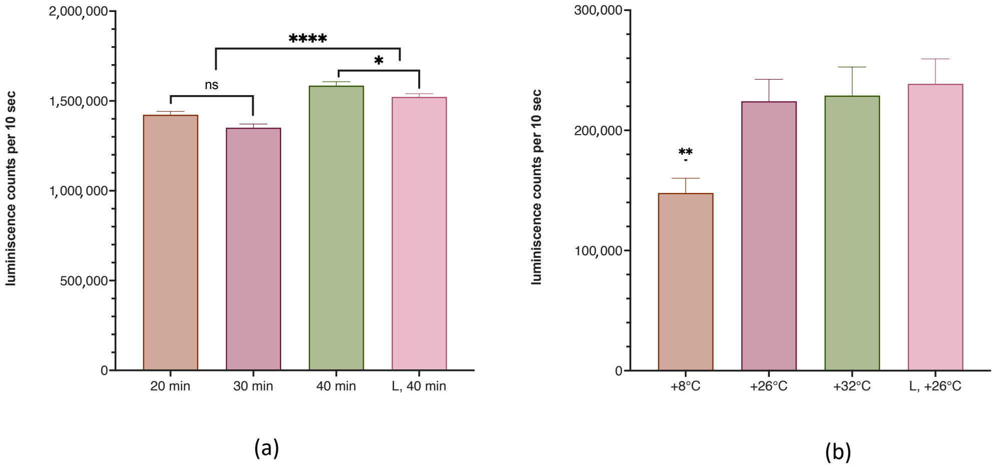

Figure S2. Transfection efficiency of HEK 293T cells depending on the conditions for the formation of LNPs (sample S1) lipoplex with mRNA. Dependence of luminescence intensity on the time (a) and temperature (b) of LNP incubation with mRNA. L – lipofectamine, * , a statistically significant difference, $p < 0.05$, **, a statistically significant difference, $p < 0.001$, ****, statistically significant difference, $p < 0.0001$, $n = 3$.

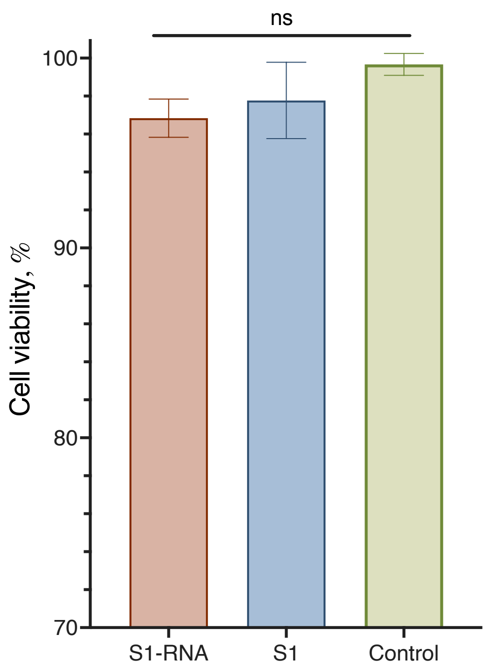

Figure S3. Cell viability upon 48h incubation of HEK263 cells with lipoplex or empty LNP. X-axis legends: S1-RNA – lipoplex LNP sample S1 with mRNA, S1 – empty LNP sample S1 (basic composition), Control – native cells, Data of one experiment with triple experimental points.

Method

Resazurin test [1]

The cytotoxicity was evaluated using a resazurin assay that is based on reducing the blue resazurin dye (7-Hydroxy-3H- phenoxazin-3-one 10-oxide) to pink colored and then highly red fluorescent resorufin by living cells. The untreated cells were used as a negative (all alive) control, and the cells treated with 50% Triton X- 100 in ethanol (3.6 µl per 200 µl of cell culture medium) were used as a positive (all dead, background) control. After a 24 h incubation, a 700 µM resazurin solution in PBS (pH 7.2) was added to the cells (10 µl per well), and the cells were transferred to a CO2-incubator for 2 h. The resorufin amount was determined using the Hidex Sense Beta Plus photometer at 594 nm with a reference wavelength of 620 nm.

1. Gretskaya, N.M.; Gamisonia, A.M.; Dudina, P.V.; Zakharov, S.S.; Sherstyanykh, G.; Akasov, R.; Burov, S.; Serkov, I.V.; Akimov, M.G.; Bezuglov, V.V.; et al. Novel Bexarotene Derivatives: Synthesis and Cytotoxicity Evaluation for Glioma Cells in 2D and 3D in Vitro Models. *Eur. J. Pharmacol.* **2020**, *883*, 173346, doi:10.1016/j.ejphar.2020.173346

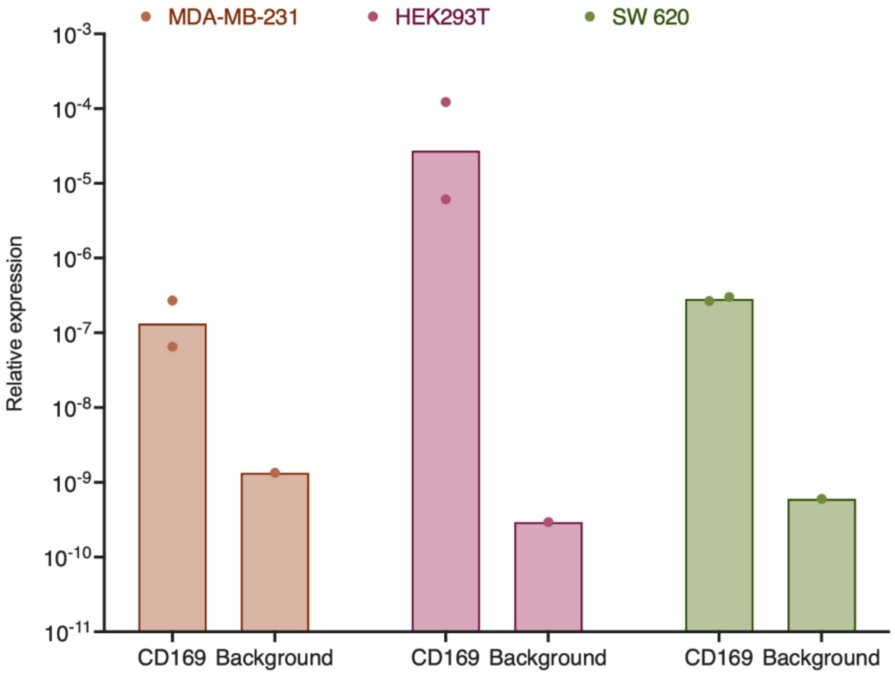

Figure S4. CD169 mRNA expression level in MDA-MB-231, SW 620, and HEK293T cell lines**.** RT-qPCR data, CD169 primer sequences: forward 3'-CACCTCCAAGTGAAGTATGCCC-5', reverse 3'-CCTGGAAGGATGTTCCTCCC-5'. The data are normalized to the expression level of the housekeeping gene rpII in each line. ($2^{-(Cq\_rpII-Cq\_cd169)}$). Background – qPCR signal without template. $n = 2$.
